# A statistical approach for 5′ splice site prediction using short sequence motifs and without encoding sequence data

**DOI:** 10.1186/s12859-014-0362-6

**Published:** 2014-11-25

**Authors:** Prabina Kumar Meher, Tanmaya Kumar Sahu, Atmakuri Ramakrishna Rao, Sant Dass Wahi

**Affiliations:** Division of Statistical Genetics, Indian Agricultural Statistics Research Institute, New Delhi, 110012 India; Centre for Agricultural Bioinformatics, Indian Agricultural Statistics Research Institute, New Delhi, 110012 India

**Keywords:** Short reads, Di-nucleotide associations, SAE, Threshold value

## Abstract

**Background:**

Most of the approaches for splice site prediction are based on machine learning techniques. Though, these approaches provide high prediction accuracy, the window lengths used are longer in size. Hence, these approaches may not be suitable to predict the novel splice variants using the short sequence reads generated from next generation sequencing technologies. Further, machine learning techniques require numerically encoded data and produce different accuracy with different encoding procedures. Therefore, splice site prediction with short sequence motifs and without encoding sequence data became a motivation for the present study.

**Results:**

An approach for finding association among nucleotide bases in the splice site motifs is developed and used further to determine the appropriate window size. Besides, an approach for prediction of donor splice sites using *sum of absolute error* criterion has also been proposed. The proposed approach has been compared with commonly used approaches *i.e.,* Maximum Entropy Modeling (MEM), Maximal Dependency Decomposition (MDD), Weighted Matrix Method (WMM) and Markov Model of first order (MM1) and was found to perform equally with MEM and MDD and better than WMM and MM1 in terms of prediction accuracy.

**Conclusions:**

The proposed prediction approach can be used in the prediction of donor splice sites with higher accuracy using short sequence motifs and hence can be used as a complementary method to the existing approaches. Based on the proposed methodology, a web server was also developed for easy prediction of donor splice sites by users and is available at http://cabgrid.res.in:8080/sspred.

**Electronic supplementary material:**

The online version of this article (doi:10.1186/s12859-014-0362-6) contains supplementary material, which is available to authorized users.

## Background

Splice sites are the regions, where introns are excised from the pre-mRNA leaving the exons alone. In general, exon-intron boundary is called donor (5′) splice site and is conserved with di-nucleotide GT whereas intron-exon boundary is called acceptor (3′) splice site and is conserved with di-nucleotide AG, together known as canonical splice sites. Approximately 99% of the splice sites are canonical GT-AG type in humans [1]. Analysis of the splice sites is very important field of computational biology due to their key role in prediction of the exon-intron structure of protein coding genes.

Recognition of splicing in short reads poses a challenge because they often align to numerous places in a genome, and often lack insufficient sequence specificity on one or both ends of exon-exon junction to accurately define junction [2]. Moreover, to utilize short reads generated from the next generation sequencing technology for transcriptome sequencing and gene structure identification, one need to align accurately the sequence reads over intron boundaries and splice site prediction helps to improve the alignment quality [3]. Therefore, it is required to develop methodology to predict splice variants using short reads or sequence with short window size.

Although, there exists methods like Weighted Matrix Method (WMM) [4], Weighted Array Model (WAM) [5], Logit linear model [6] etc. for the prediction of splice sites, most of the methods are based on Classification tree [7-9], Artificial Neural Networks (ANNs) [10-13] and Support Vector Machines (SVMs) [14-21]. In splice site prediction using Machine Learning Approaches (MLAs) like ANN and SVM, initially the splice sites are encoded into numeric form and then the encoded data is used as input for prediction [19,22,23]. Although, most of these methods have shown high prediction accuracy *i.e.,* >90%, the lengths of window sizes used are longer *i.e.*, more than 40 base pairs (bp) [16,20,22,23]. Hence, these approaches may not be suitable for predicting splice variants using short sequence reads generated from next generation sequencing technologies.

In the present study, an attempt has been made to develop a method for the prediction of donor splice sites, using shorter window size, based on the idea of di-nucleotide association. The proposed method can be used to predict the donor splice sites without encoding sequence data usually required in MLAs. The process of sequence data encoding is one step more for the prediction that may require additional memory/storage allocation. Besides, the results (prediction accuracy) also vary with different encoding approaches. The proposed method was also compared with the commonly used methods that are based on short sequences and without encoding the sequence data.

## Methods

### Data

True Splice Site (TSS) and False Splice Site (FSS) sequences were collected from Homo Sapiens Splice Site Dataset (HS3D) available at URL: (http://www.sci.unisannio.it/docenti/rampone/) [24]. The collected dataset contains 2796 TSS and 90924 FSS sequences corresponding to 622 and 125 genes respectively. The length of each sequence is 140 bp having 70 bp on both the exon and intron side with conserved GT at 71^st^ and 72^nd^ positions. From the collected data, it was found that the maximum number of TSS present in a gene is around 50 whereas the maximum number of FSS is around 8000, which implies imbalanced-ness between the presence of TSS and FSS in a gene.

### Associations among nucleotides

Here, we propose an approach for finding associations among nucleotides in the splice site motifs and is explained as follows:

Consider a sequence dataset having *N* sequences of equal length *P* and let *S*_*k*_ = (*x*_1*k*_, *x*_2*k*_, …, *x*_*Pk*_), *x*_*ik*_ ∈ {*A*, *T*, *G*, *C*} ; ∀ *i* = 1, 2, …, *P* be the *k*^th^ sequence. Then for the *i*^th^ position, the occurrence of base *s* is described by an indicator variable *I*_*is*_*i.e.,*

$$ {I}_{is}=I\left({x}_{ik}=s\right)=\left\{\begin{array}{l}1,\kern0.24em \mathrm{if}\;s\kern0.24em \mathrm{occurs}\\ {}0,\kern0.24em \mathrm{otherwise}\end{array}\right.\kern0.36em \forall i=1,2,\dots, P;\kern0.24em s\kern0.48em \in \left\{A,T,G,C\right\};\kern0.36em k=1,2,\dots, N $$

Now, the proportion of base *s* occurring at the *i*^th^ position is given by

$$ p\left({X}_i=s\right)=\frac{1}{N}{\displaystyle \sum_{k=1}^NI\left({x}_{ik}=s\right)};\kern0.48em s\kern0.48em \in \left\{A,T,G,C\right\} $$

and the proportion of base *s* and *t* occurring together at *i*^th^ and *j*^th^ position respectively is given by

$$ p\left({X}_i=s,\;{X}_j=t\right)=\frac{1}{N}{\displaystyle \sum_{k=1}^NI\left({x}_{ik}=s\right)I\left({x}_{jk}=t\right)}\kern0.24em ;\kern0.6em s,t\in \left\{A,T,G,C\right\} $$

The association between base *s* and *t* at *i*^th^ and *j*^th^ position respectively is then computed as

1 $$ {a}_{i,j}\left(s,t\right)=\frac{{\displaystyle \sum_{k=1}^NI\left({x}_{ik}=s\right)I\left({x}_{jk}=t\right)}}{\sqrt{\;{\displaystyle \sum_{k=1}^NI\left({x}_{ik}=s\right)}\;.\kern0.24em {\displaystyle \sum_{k=1}^NI\left({x}_{jk}=t\right)}}}\forall i\ne j $$

The range of this association lies between 0 (no association) and 1 (complete association). A “no association” situation occurs when base *s* at *i*^th^ position and base *t* at *j*^th^ position do not occurs together in any of the *N* sequences *i.e.,*${\displaystyle \sum_{k=1}^NI\left({x}_{ik}=s\right)I\left({x}_{jk}=t\right)}=0$ and hence *a*_*i*,*j*_(*s*, *t*) = 0. On the other hand, whenever *s* occurs at *i*^th^ position, *t* occurs at *j*^th^ position correspondingly in all the *N* sequences then there exist a complete association between *s* and *t*, *i.e.,*${\displaystyle \sum_{k=1}^NI\left({x}_{ik}=s\right)I\left({x}_{jk}=t\right)}={\displaystyle \sum_{k=1}^NI\left({x}_{ik}=s\right)}={\displaystyle \sum_{k=1}^NI\left({x}_{jk}=t\right)}=C(say)$ and hence ${a}_{i,j}\left(s,t\right)=\frac{C}{\sqrt{\;C\;.\kern0.24em C}}=1$.

However, for *i* = *j*, the association between base *s* and *t* can be calculated using the formula

2 $$ {a}_{i,i}\left(s,t\right)=\left\{\begin{array}{l}-\sqrt{\frac{{\displaystyle \sum_{k=1}^NI\left({x}_{ik}=s\right)}\;.\kern0.24em {\displaystyle \sum_{k=1}^NI\left({x}_{ik}=t\right)}}{\left(N-{\displaystyle \sum_{k=1}^NI\left({x}_{ik}=s\right)}\right)\;.\kern0.24em \left(N-{\displaystyle \sum_{k=1}^NI\left({x}_{ik}=t\right)}\right)}}\kern0.72em \mathrm{if}\kern0.36em s\ne t\\ {}1\kern11.64em \mathrm{if}\kern0.48em s=t\end{array}\right. $$

following the theory of multinomial distribution. Here, every position can be modeled empirically with a tetranomial (multinomial with four different outcomes corresponding to nucleotides A, T, G and C) distribution, where the probability of success of each outcome can be computed empirically from the position-wise aligned sequence data as $p\left({s}_i\right)=\frac{1}{N}{\displaystyle \sum_{k=1}^NI\left({x}_{ik}=s\right)}$, where *p*(*s*_*i*_) is the probability of the outcome *s* at *i*^th^ position; *i* = 1, 2, …, *P* and *s* ∈ {*A*, *T*, *G*, *C*}.

More elaborately, the association matrix between base *s* and *t* occurring at *i*^th^ and *j*^th^ positions respectively in splice site sequences can be constructed as;

$$ \left(\begin{array}{ccccc}\hfill {a}_{1,1}\left(s,\ t\right)\hfill & \hfill \dots \hfill & \hfill {a}_{1,j}\left(s,\ t\right)\hfill & \hfill \dots \hfill & \hfill {a}_{1,P}\left(s,\ t\right)\hfill \\ {}\hfill \vdots \hfill & \hfill\ \hfill & \hfill \vdots \hfill & \hfill\ \hfill & \hfill \vdots \hfill \\ {}\hfill {a}_{i,1}\left(s,\ t\right)\hfill & \hfill \dots \hfill & \hfill {a}_{i,j}\left(s,\ t\right)\hfill & \hfill \dots \hfill & \hfill {a}_{i,P}\left(s,\ t\right)\hfill \\ {}\hfill \vdots \hfill & \hfill\ \hfill & \hfill \vdots \hfill & \hfill\ \hfill & \hfill \vdots \hfill \\ {}\hfill {a}_{P,1}\left(s,\ t\right)\hfill & \hfill \dots \hfill & \hfill {a}_{P,j}\left(s,\ t\right)\hfill & \hfill \dots \hfill & \hfill {a}_{P,P}\left(s,\ t\right)\hfill \end{array}\right) $$

where, the off-diagonal and diagonal elements of the matrix will be obtained by using the equation (1) and (2) respectively.

The prediction approach is based on the di-nucleotide dependencies at all possible pairs of positions for a given window size and that became motivation for determining the window size on the basis of di-nucleotide association. Thus, the said association measure was introduced. The proposed association measure seems to be more informative than the existing position wise measures because (i) in the position-wise association (or mutual information) [25] only a single observation is obtained between any two positions whereas in the proposed association measure there will be 16 observations between any two positions and (ii) the positional level associations are the function of nucleotide level associations. This approach can also be used for finding the associations in any position-wise aligned sequence dataset having sequences of equal length, provided the number of sequences in the dataset is large *i.e.,* the probability of occurrence of any base at any position should be non-zero.

### Splice site prediction approach

The value (1, 0) of the indicator variable *I*_*is*_ (defined in the previous sub-section) is considered as the observed value for the base *s* at *i*^th^ position in the motif and the estimated value of base *s* at *i*^th^ position given base *t* at *j*^th^ position is computed as *E*(*I*_*is*_|*I*_*jt*_), where *j* ≠ *i*. Since the expectation of an indicator variable is nothing but probability, *E*(*I*_*is*_|*I*_*jt*_) = *p*(*s*_*i*_|*t*_*j*_) ∀ *j* ≠ *i* ; *s*, *t* ∈ {*A*, *T*, *G*, *C*}, which is the proportion of base *s* at *i*^th^ position given base *t* at *j*^th^ position. More elaborately,

$$ E\left({I}_{is}\Big|{I}_{jt}\right)=p\left({s}_i\Big|{t}_j\right)=\frac{{\displaystyle \sum_{k=1}^NI\left({x}_{ik}=s\right)I\left({x}_{jk}=t\right)}}{{\displaystyle \sum_{k=1}^NI\left({x}_{jk}=t\right)}} $$

Now, the Sum of Absolute Error (SAE) for the *i*^th^ position is computed as

$$ \begin{array}{l}{\mathrm{SAE}}_i={\displaystyle \sum_{j=1;j\ne i}^P\left|1-p\left({s}_i\Big|{t}_j\right)\right|+\left|0-\left(1-p\left({s}_i\Big|{t}_j\right)\right)\right|}\\ {}\kern0.6em =2{\displaystyle \sum_{j=1;j\ne i}^P\left\{1-p\left({s}_i\Big|{t}_j\right)\right\}}=2\left(P-1\right)-2{\displaystyle \sum_{j=1;j\ne i}^Pp\left({s}_i\Big|{t}_j\right)}\end{array} $$

Hence, the SAE over all positions (SAE_ap_) for the sequence of length *P* is given by ${\mathrm{SAE}}_{\mathrm{ap}}={\displaystyle \sum_{i=1}^P{\mathrm{SAE}}_{\mathrm{i}}}=2{\displaystyle \sum_{i=1}^P\left[{\displaystyle \sum_{j=1;j\ne i}^P\left\{1-p\left({s}_i\Big|{t}_j\right)\right\}}\right]}=2P\left(P-1\right)-2{\displaystyle \sum_{i=1}^P{\displaystyle \sum_{j=1;j\ne i}^Pp\left({s}_i\Big|{t}_j\right)}}$

#### Prediction of test instance

The following steps are followed for prediction of a test instance

1. Compute the SAE of the test instance by assuming it as TSS (${\mathrm{SAE}}_{ap}^T$) *i.e.,* the SAE_*ap*_ of the test instance will be calculated by using the conditional probability *p*(*s*_*i*_|*t*_*j*_) based on the training dataset of TSS.
2. Compute the SAE of the test instance by assuming it as FSS (${\mathrm{SAE}}_{ap}^F$) *i.e.,* the SAE_*ap*_ of the test instance will be calculated by using the conditional probability *p*(*s*_*i*_|*t*_*j*_) based on the training dataset of FSS.
3. Compute the difference *i.e.,*${\mathrm{dSAE}}_{ap}^{T-F}={\mathrm{SAE}}_{ap}^T-{\mathrm{SAE}}_{ap}^F={\left\{2{\displaystyle \sum_{i=1}^P{\displaystyle \sum_{j=1;j{}^1i}^Pp\left({s}_i\Big|{t}_j\right)}}\right\}}^T-{\left\{2{\displaystyle \sum_{i=1}^P{\displaystyle \sum_{j=1;j{}^1i}^Pp\left({s}_i\Big|{t}_j\right)}}\right\}}^F$
4. $$ \left\{\begin{array}{l} if\kern0.24em {\mathrm{dSAE}}_{ap}^{T-F}<\varepsilon, \kern0.6em \mathrm{the}\kern0.24em \mathrm{instance}\kern0.24em \mathrm{is}\kern0.24em \mathrm{predicted}\kern0.24em \mathrm{a}\mathrm{s}\;\mathrm{T}\mathrm{S}\mathrm{S}\\ {} if\kern0.24em {\mathrm{dSAE}}_{ap}^{T-F}\ge \varepsilon, \kern0.6em \mathrm{the}\kern0.24em \mathrm{instance}\kern0.24em \mathrm{is}\kern0.24em \mathrm{predicted}\kern0.24em \mathrm{a}\mathrm{s}\;\mathrm{F}\mathrm{S}\mathrm{S}\end{array}\right.\kern0.36em ;\kern0.48em \varepsilon \in R $$

For estimating the threshold value (*ε*), the following steps are followed:

I. Take a random data set (containing 60% of observations) from the original data set and divide it into 10 non-overlapping sets with each set containing approximately same number of TSS and FSS.
II. Everytime, use one set of TSS and FSS together as a test set and remaining nine sets of TSS and FSS together as a training set.
III. Calculate the performance accuracy in terms of *sensitivity* and *specificity* using the test dataset.
IV. Calculate the performance accuracy for different threshold values for each test set and retain the value of threshold where *specificity* = *sensitivity*.
V. Obtain the final threshold value by taking the average of the threshold values over ten test sets.

### Heat map generation

All the sequences in TSS and FSS were used to generate the association matrices, where the length of each sequence used was 20 bp having 10 bp on both side of conserved di-nucleotide GT at the beginning of the intron. The sequence length of 20 bp was considered initially to have an idea on the associations among nucleotides at splice sites. However, the sequence length can be increased if the association patterns are expected beyond the considered sequence length.

The association matrices were obtained for all the 16 di-nucleotide combinations separately for TSS and FSS. Out of these, only 10 combinations *i.e.,* AA, AT, AG, AC, TT, TG, TC, GG, GC and CC are required to fully portrait the association structure and the remaining 6 associations *i.e.,* TA, GA, GT, CA, CT and CG can be obtained by taking the transpose of the associations AT, AG, TG, AC, TC and GC respectively. For example, the association matrix generated using association between G and T is the transpose of the association matrix generated using association between T and G. The association matrices obtained from TSS and FSS were merged separately and heat maps were generated using the *stats* package of R-software to visualize the association pattern. The association pattern was used to determine the window size.

### Redundancy check and similarity search

A redundancy check was performed on the dataset with the determined window size to remove the duplicate sequences (100% identical) as non-removal of such sequences may lead to biasness (in terms of prediction accuracy) towards the class having larger proportion of duplicate sequences. The duplicate sequences within TSS and FSS were removed first and then the sequences present in TSS were removed from the FSS.

After removing the duplicate sequences, sequence distribution was analyzed by performing a similarity search (using a developed R-code), where each sequence of TSS was compared with the other sequences of TSS as well as with all the sequences of FSS and vice versa. The percentage of similarity between any two sequences was calculated by assigning a score of 1 and 0 for every match and mismatch in nucleotides respectively and the same is explained below for two sample sequences.

- Sequence 1: ATTCGTCATG
- Sequence 2: TCTAGTTACG
- Score : 0010110101
- Similarity (%)=(5/10)*100=50

The necessity of similarity search lies in the fact that if there exists similarity within TSS & FSS and the sequences of TSS are completely distinct from the sequences of FSS (*i.e.,* zero similarity) then it is obvious that the classification accuracy by using such datasets as training and test set will be greater. However, the TSS and FSS sequences occur in the nature are not completely distinct from each other. Therefore, there should be similarity between the sequences of TSS and FSS dataset to judge the actual predictive ability of the prediction method.

### Performance comparison using HS3D dataset

The dataset with the determined window size, obtained after redundancy check, was used to compare the performance of the proposed approach with that of existing score based approaches, *viz.,* Maximum Entropy Model (MEM) score [26], Maximal Dependency Decomposition (MDD) score [7], Weighted Matrix Method (WMM) score [4] and Markov model of first order (MM1) score. The comparison was made using Receiving Operating Characteristics (ROC) curves, Precision-Recall (PR) curves, estimates of Area Under ROC curves (AUC-ROC) and Area Under PR curves (AUC-PR). For the purpose of comparison, the scores of MEM, MDD, WMM and MM1 were obtained by executing the MaxEntScan (a web server) using the considered dataset. The web server is available at http://genes.mit.edu/burgelab/maxent/Xmaxentscan_scoreseq.html.

### ROC and PR analysis

An ROC graph depicts the relative trade-offs between true positives and false positives. It compares the classifiers' performance across the entire range of class distributions and error costs. To measure the performance accuracy of the proposed approach and to compare it with the existing approaches, ROC curves were plotted and the AUC-ROC values were also computed. Further, the statistical comparison between two ROC curves was made by using the Standard Error (SE) of AUC-ROC [27], which was computed as

$$ \mathrm{S}\mathrm{E}=\sqrt{\frac{\uptheta \left(1\hbox{-} \uptheta \right)+\left({\mathrm{N}}^{\left(\mathrm{T}\mathrm{S}\mathrm{S}\right)}\hbox{-} 1\right)\left({\mathrm{Q}}_1\hbox{-} {\uptheta}^2\right)+\left({\mathrm{N}}^{\left(\mathrm{F}\mathrm{S}\mathrm{S}\right)}\hbox{-} 1\right)\left({\mathrm{Q}}_2\hbox{-} {\uptheta}^2\right)}{{\mathrm{N}}^{\left(\mathrm{T}\mathrm{S}\mathrm{S}\right)}.{\mathrm{N}}^{\left(\mathrm{F}\mathrm{S}\mathrm{S}\right)}}}, $$

where ${\mathrm{Q}}_1=\frac{\uptheta}{\left(2\hbox{-} \uptheta \right)}\kern0.72em \mathrm{and}\kern0.36em {\mathrm{Q}}_2=\frac{2.{\uptheta}^2}{\left(1+\uptheta \right)}$ and N^(TSS)^, N^(FSS)^ and θ are the number of positive instances (TSS), number of negative instances (FSS) and estimate of AUC-ROC respectively.

Since AUC-ROC is invariant to the class-skew, it is not an appropriate measure under imbalanced data situation and hence in addition to the AUC-ROC, PR curves and AUC-PR were also used for evaluating the performance. PR curves were obtained by taking Recall on the X-axis and Precision on the Y-axis, where the correct Recall-Precision points were obtained by using the interpolation technique suggested by Davis and Goadrich [28]. The interpolation technique is described as follows:

Let A and B be two points, which are far apart in Precision-Recall space and is generated from true positive (TP_A_) and false positive (FP_A_) counts, where TP_A_ ≤ TP_B_ and FP_A_ ≤ FP_B_. Then, interpolation is done between the counts TP_A_ and TP_B_, and FP_A_ and FP_B_ to create intermediate points between A and B. The intermediate TP counts are created as TP_A_ + 1; TP_A_ + 2; …;TP_B_ -1 and corresponding FP are obtained by linearly increasing the false positives for each new point by the local skew $\left(\frac{{\mathrm{FP}}_{\mathrm{B}}-{\mathrm{FP}}_{\mathrm{A}}}{{\mathrm{TP}}_{\mathrm{B}}-{\mathrm{TP}}_{\mathrm{A}}}\right)$.

### Evaluation with varying window sizes

To be more confident with the determined window size, the performance of the proposed approach was also analyzed with other window sizes in addition to the determined window size. The redundancy checks were also performed for different window sizes, in the similar way as described earlier.

### Evaluation using imbalanced dataset

To assess the performance of the proposed approach with respect to different degrees of imbalanced-ness, along with the balanced dataset three more datasets were prepared containing TSS and FSS in the proportions of 1:2.5, 1:5 and 1:7.5 respectively. The proposed approach was executed using these datasets and the performances were assessed by plotting the ROC and PR curves using the results obtained from the 10-fold cross validation technique.

### Performance comparison using DGSplicer dataset

To check the consistency of the proposed prediction approach, a comparison was also made with the other considered approaches using the bench mark DGSplicer dataset available at URL: http://www.fruitfly.org/data/seq_tools/datasets/Human/GENIE_96/splicesets/. The collected dataset contains 2359 TSS and equal number of FSS with window size of 9 bp long. Comparison between the proposed approach and other considered approaches was made using ROC curves, PR curves, estimates of AUC-ROC and AUC-PR.

### Evaluation with redundant test dataset

In addition to the performance evaluation using non-redundant test dataset, the performance of the proposed approach along with the other approaches were also evaluated using the test dataset having redundant sequences. To evaluate the performance, 4 datasets are prepared in which one is balanced and other three are imbalanced. The performances were assessed in terms of AUC-ROC and AUC-PR.

### Web server

A web interface was developed to help the biological community for the prediction of donor splice sites using the developed approach. It was developed using HTML and PHP, where the developed R-code was executed in the background upon the submission of a single or multiple nucleotide sequences in FASTA format. To submit the sequence(s), the facilities for both pasting the sequence(s) in a text area and uploading a FASTA file are provided. The processed results are displayed in the same page and a link is provided for downloading the original result file.

## Results

### Heat maps and window size

**Figure 1:**
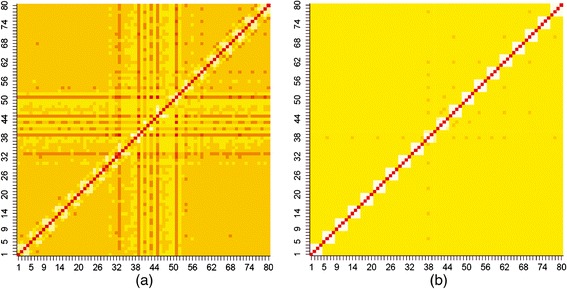
**Heat map of TSS and FSS.** Heat maps of **(a)** TSS and **(b)** FSS were generated by using corresponding association matrices. Association matrices were generated by taking 20 positions (10 positions at the exon end and 10 positions excluding GT at the intron start). Since each position corresponds to four indicator variables, hence the heat map generated is of order 80 × 80 units and the units between 29–40 indicates 3 bp at the exon end and 41–64 units for 6 bp at the intron start. There exist distinct association pattern among the positions around the conserved di-nucleotide GT in TSS. On the other hand, such association pattern is absent in case of FSS.

From the heat map of TSS (Figure 1a), it is seen that the positions in the signal region are associated with each other and the positions away from the signal region have association with the positions in the signal region as well. Further, it is observed that most of the associations are found between 29–64 units (each unit correspond to the occurrence of one nucleotide at a given position *i.e.,* every position will have 4 units), which corresponds to position number 8–16 out of considered 20 positions in the motif. On the other hand, from the heat map of FSS (Figure 1b), it is noticed that no such association pattern among the positions is present. Taking the above association pattern into consideration, the window size determined was of length 9 bp.

### Redundancy and similarity analysis

**Figure 2:**
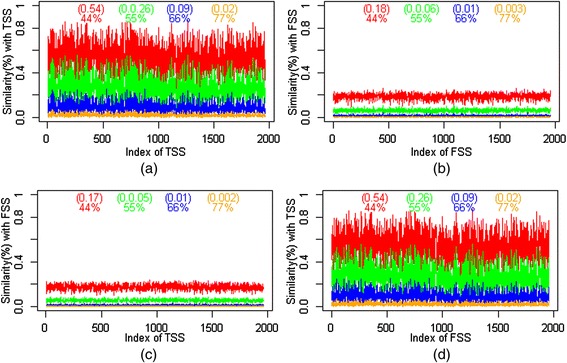
**Percentage of similarity within and between TSS and FSS.** It shows different percentages of similarity that exists **(a)** within TSS **(b)** within FSS **(c)** TSS with FSS **(d)** FSS with TSS. The value inside parenthesis indicates the proportion of similar sequences with corresponding percentage of similarity (same color) shown below the parenthesis. It can be noticed that there exist a maximum of 77% similar sequences within and between TSS and FSS.

The redundant sequences were removed from the resulting data set of 9 bp window size and a total number of 1960 unique TSS and 59097 unique FSS were obtained. The similarity search performed on the unique TSS and FSS (number of FSS are kept equal to the number of TSS and are drawn at random from the available unique FSS) datasets revealed that at most 77% similarity exists within and between TSS and FSS. It is observed that each sequence of TSS shows 77% (orange) similarity with on an average 39 (2% of 1960) sequences of TSS (Figure 2a) and 4 (0.02% of 1960) sequences of FSS (Figure 2c). On the other hand, each sequence of FSS shows 77% (orange) similarity with on an average 6 (0.03% of 1960) sequences of FSS (Figure 2b) and 39 (2% of 1960) sequences of TSS (Figure 2d). This implies the existence of similarity among sequences within and between the classes.

In addition to the determined 9 bp window size, four more window sizes of lengths 7 bp, 8 bp, 10 bp and 11 bp were also considered in the vicinity of the splice junction to assess the effect of window sizes on prediction accuracy. The number of non-redundant sequences obtained from the TSS and FSS datasets with the above window sizes is given in Table 1.

**Table 1:** **Threshold values and estimates of AUC-ROC for the proposed approach under different window sizes**

**Different window sizes**	**7 bp**	**8 bp**	**9 bp**	**10 bp**	**11 bp**
	**(−3 ~ +4)**	**(−2 ~ +6)**	**(−3 ~ +6)**	**(−3 ~ +7)**	**(−4 ~ +7)**
**Number of TSS**	770	1370	1960	2342	2530
**Number of FSS**	12805	33840	59097	74140	88346
**Threshold (** ***ε*** **)**	[−4.00]	[−5.63]	[−7.16]	[−8.83]	[−9.97]
**AUC-ROC**	92.74	93.37	94.43	94.39	93.87
**SE**	0.006	0.004	0.003	0.003	0.003

(−) indicates from exon side and (+) indicate from intron side excluding GT. Values in the square brackets are the final threshold values.

### Performance with balanced data

**Figure 3:**
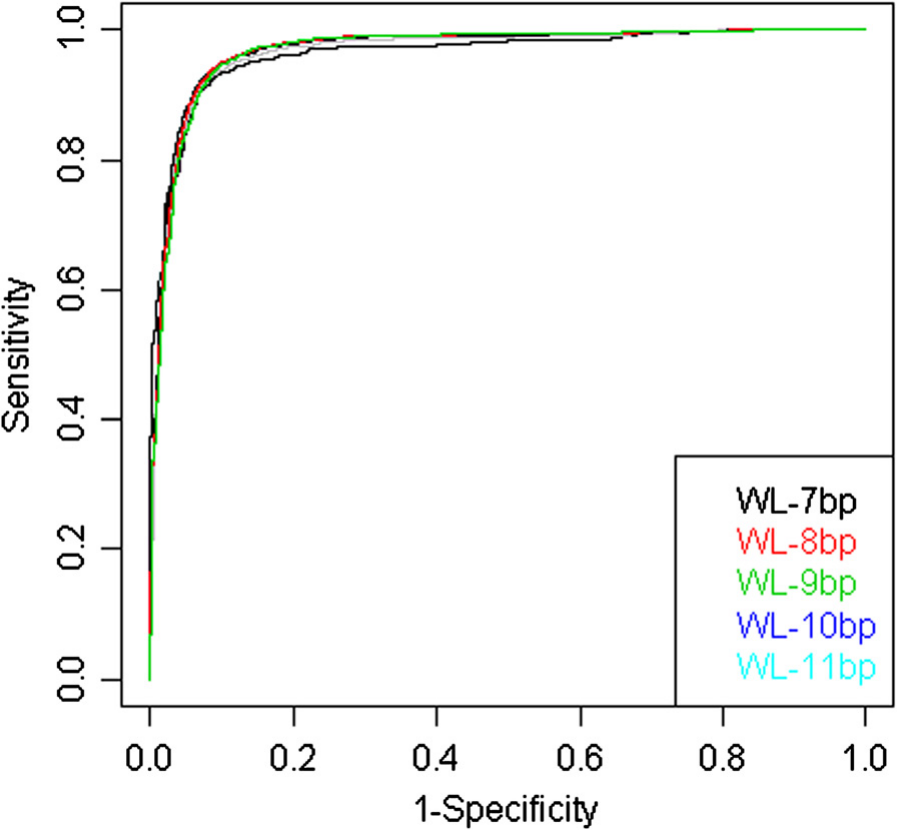
**ROC curves for the proposed approach under balanced situation with different window length (WL).**

The threshold value for the 9 bp window size was obtained as −7.16 (for details, see Additional file 1) and threshold values for the other window sizes are obtained in similar way (Table 1). The performance accuracy of the proposed approach for different window sizes with the balanced dataset is shown in the Figure 3 and Table 1 in terms of ROC and AUC-ROC respectively. For the balanced dataset, same numbers of FSS as TSS were drawn at random from the unique FSS dataset. For example, in case of 7 bp window size the number of unique TSS are 770 and unique FSS are 12805. So, 770 unique FSS need to be drawn at random from the dataset of 12805 unique FSS to get a balanced dataset. Though from Figure 3 it is difficult to choose the better ROC curve, it is observed that the estimate of AUC-ROC is highest for 9 bp window size as compared to the others (Table 1).

### Performance with imbalanced data

**Figure 4:**
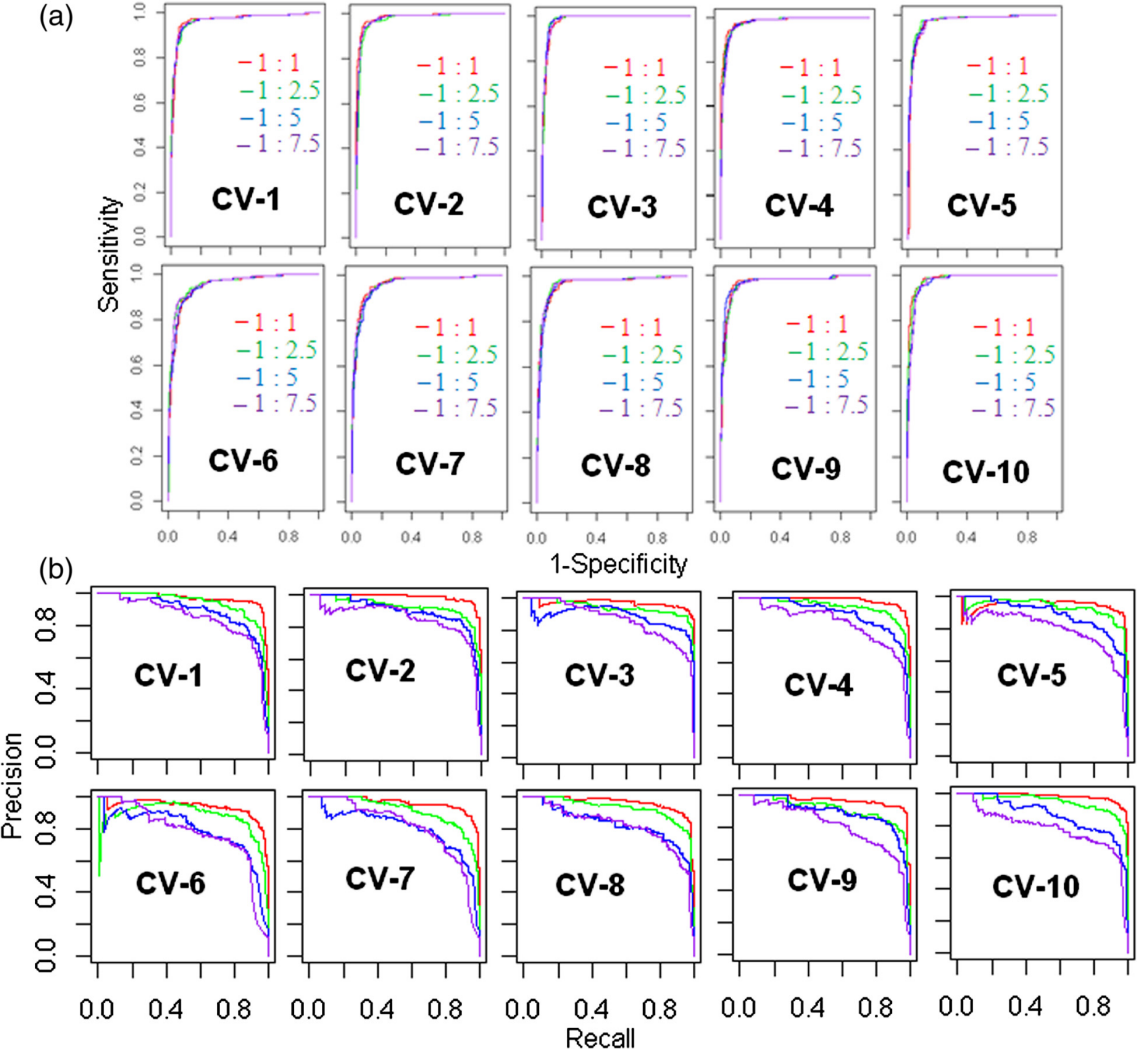
**ROC and PR curves for the proposed prediction approach. (a)** ROC curves and **(b)** PR curves are plotted using sensitivity and specificity, obtained from the test sets of 10-fold cross validation, under different degrees of imbalanced-ness. Red color curve denotes the curve for the balanced data. Green, blue and purple are the curves for the dataset with different degrees of imbalanced-ness indicated as legend. Legends for PR curves are same as the legends for ROC curves.

Since 9 bp window size is found more preferable window size under balanced situation, the same size was considered for the imbalanced situation also. The total number of TSS and FSS with different proportion, as discussed in the implementation section, is given in Table 2. Here, the numbers of FSS were drawn at random from the dataset of 59097 unique FSS. The performance of the proposed approach with respect to imbalanced dataset was assessed using 10-fold cross validation technique. The threshold values were remain same for the dataset with different degrees of imbalanced-ness under a given window size because the SAE values were calculated class wise and sensitivity, specificity used to determine the threshold values are invariant to class skew (for details, see Additional file 1). From the ROC curves (Figure 4a), it is seen that the AUC-ROC is almost same for the dataset with different degrees of imbalanced-ness as AUC-ROC is invariant to class skew. However, from the PR curves (Figure 4b) the performance of the proposed approach under balanced situation seems to be better than that of imbalanced situation and this may be due to the fact that the performance of a random guesser is equals the fraction of positive data points in an imbalanced dataset having large number negative data points as compared to positive data points.

**Table 2:** **Number of non-redundant TSS and FSS sequence under different degrees of imbalanced-ness**

**Proportion of TSS and FSS**	**Number of TSS**	**Number of FSS**
1:1	1960	1960
1:2.5	1960	5000
1:5	1960	10000
1:7.5	1960	15000

The numbers of FSS are not exact values but they are approximated to nearby integer.

### Comparative analysis using non-redundant HS3D dataset

**Figure 5:**
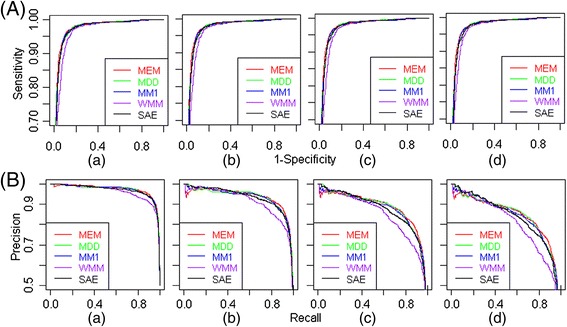
**ROC and PR curves for different splice site prediction approaches using HS3D dataset. (A)** ROC curves and **(B)** PR curves for the proposed (SAE) and other considered approaches in prediction of donor splice sites are plotted for (a) balanced dataset and imbalanced dataset having unequal number TSS and FSS *i.e.,* (b) 1960 & 5000, (c) 1960 & 10000 and (d) 1960 & 15000 respectively.

The performance of the proposed approach is compared with the existing approaches using ROC curves (Figure 5A), PR curves (Figure 5B), estimates of AUC-ROC and AUC-PR (Table 3). It is observed that the values of AUC-ROC for MEM, MDD and SAE are almost same under both balanced and imbalanced situation and higher than that of WMM and MM1. It is further observed that the values of AUC-PR for MEM and SAE are at par.

**Table 3:** **Estimates of AUC-ROC and AUC-PR for the proposed approach and other considered approaches (non-redundant case)**

	**AUC-ROC**				**AUC-PR**			
**Approaches**	**A**	**B**	**C**	**D**	**A**	**B**	**C**	**D**
**MEM**	0.946	0.947	0.947	0.945	0.946	0.868	0.76	0.675
	(±0.0036)	(±0.0035)	(±0.0035)	(±0.0036)	(±0.0036)	(±0.0054)	(±0.0065)	(±0.0069)
**MDD**	0.945	0.945	0.944	0.943	0.939	0.864	0.759	0.668
	(±0.0037)	(±0.0036)	(±0.0036)	(±0.0037)	(±0.0039)	(±0.0055)	(±0.0066)	(±0.0070)
**MM1**	0.940	0.940	0.937	0.937	0.937	0.863	0.749	0.666
	(±0.0038)	(±0.0038)	(±0.0038)	(±0.0038)	(±0.0038)	(±0.0055)	(±0.0067)	(±0.0070)
**WMM**	0.922	0.924	0.921	0.922	0.917	0.825	0.686	0.585
	(±0.0045)	(±0.0043)	(±0.0042)	(±0.0042)	(±0.0044)	(±0.0061)	(±0.0070)	(±0.0071)
**SAE**	0.944	0.944	0.944	0.943	0.944	0.867	0.761	0.673
	(±0.0037)	(±0.0037)	(±0.0036)	(±0.0037)	(±0.0037)	(±0.0054)	(±0.0066)	(±0.0069)

A - Balanced, B- Imbalanced-I, C- Imbalanced-II, D- Imbalanced-III.

The values inside the parentheses are the Standard Errors.

### Comparative analysis using redundant DGSplicer dataset

**Figure 6:**
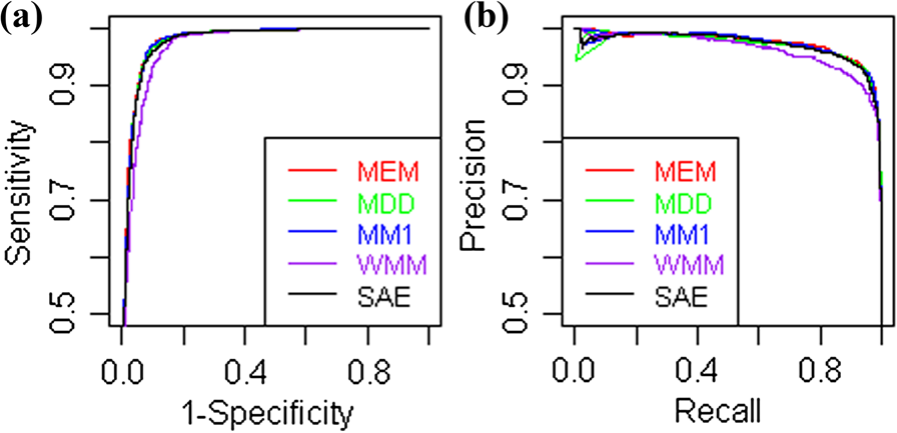
**ROC and PR curves for the proposed approach and other considered approaches using DGSplicer dataset. (a)** ROC curves and **(b)** PR curves for different approaches are plotted using the DGSplicer dataset as the test dataset.

The ROC and PR curves for the DGSplicer dataset are plotted in Figure 6 and AUC-ROC, AUC-PR is presented in Table 4. From the table it is observed that the AUC-ROC for SAE and MDD are almost same and is very close to that of MEM. However, AUC-PR is almost similar for SAE and MEM and is slightly better than that of MDD. After looking at the overall performances, it is inferred that the SAE, MEM and MDD are performing at par and are better than the WMM and MM1.

**Table 4:** **Estimate of AUC-ROC and AUC-PR for the DGSplicer dataset using different approaches**

**Approaches**	**AUC-ROC ± SE**	**AUC-PR ± SE**
**MEM**	0.957 ± 0.0030	0.948 ± 0.0033
**MDD**	0.956 ± 0.0030	0.940 ± 0.0034
**MM1**	0.954 ± 0.0031	0.938 ± 0.0036
**WMM**	0.936 ± 0.0037	0.923 ± 0.0040
**SAE**	0.956 ± 0.0030	0.947 ± 0.0033

### Performance analysis with and without redundancy in HS3D test dataset

The number of TSS and FSS sequence along with the number of redundant sequence present in the redundant test dataset is given in Table 5. After looking at the AUC-ROC and AUC-PR (Table 6), it can be said that the performance of the approaches are better in case of redundant test dataset as compared to the non-redundant test dataset (dataset mentioned in Table 2).

**Table 5:** **Number of redundant sequences present in the HS3D dataset considered for evaluation of proposed approach**

**Type of data**	**Total number of sequences (TSS, FSS)**	**Number of redundant sequences (TSS, FSS)**
**Balanced**	(2796,2796)	(830, 102)
**Imbalanced-I**	(2796,5000)	(830, 231)
**Imbalanced-II**	(2796,10000)	(830,828)
**Imbalanced-III**	(2796,15000)	(830,1727)

**Table 6:** **Estimates of AUC-ROC and AUC-PR of different approaches executed using redundant test dataset**

	**AUC-ROC**				**AUC-PR**			
**Approaches**	**A**	**B**	**C**	**D**	**A**	**B**	**C**	**D**
**MEM**	0.948	0.946	0.947	0.947	0.947	0.878	0.773	0.683
	(±0.0031)	(±0.0031)	(±0.0030)	(±0.0030)	(±0.0031)	(±0.0045)	(±0.0055)	(±0.0059)
**MDD**	0.945	0.942	0.944	0.944	0.944	0.872	0.769	0.680
	(±0.0031)	(±0.0032)	(±0.0030)	(±0.0030)	(±0.0031)	(±0.0046)	(0.0055)	(±0.0059)
**MM1**	0.945	0.941	0.936	0.941	0.942	0.870	0.765	0.679
	(±0.0031)	(±0.0032)	(±0.0032)	(±0.0031)	(±0.0032)	(±0.0046)	(±0.0056)	(±0.0060)
**WMM**	0.927	0.924	0.924	0.925	0.924	0.867	0.703	0.675
	(±0.0036)	(±0.0036)	(±0.0035)	(±0.0034)	(±0.0037)	(±0.0046)	(±0.0060)	(±0.0060)
**SAE**	0.946	0.945	0.944	0.945	0.945	0.876	0.772	0.682
	(±0.0031)	(±0.0031)	(±0.0030)	(±0.0030)	(±0.0031)	(±0.0045)	(±0.0055)	(±0.0059)

A- Balanced, B- Imbalanced-I, C- Imbalanced-II, D- Imbalanced-III.

The values inside the parentheses are Standard Errors.

### A user friendly web interface

**Figure 7:**
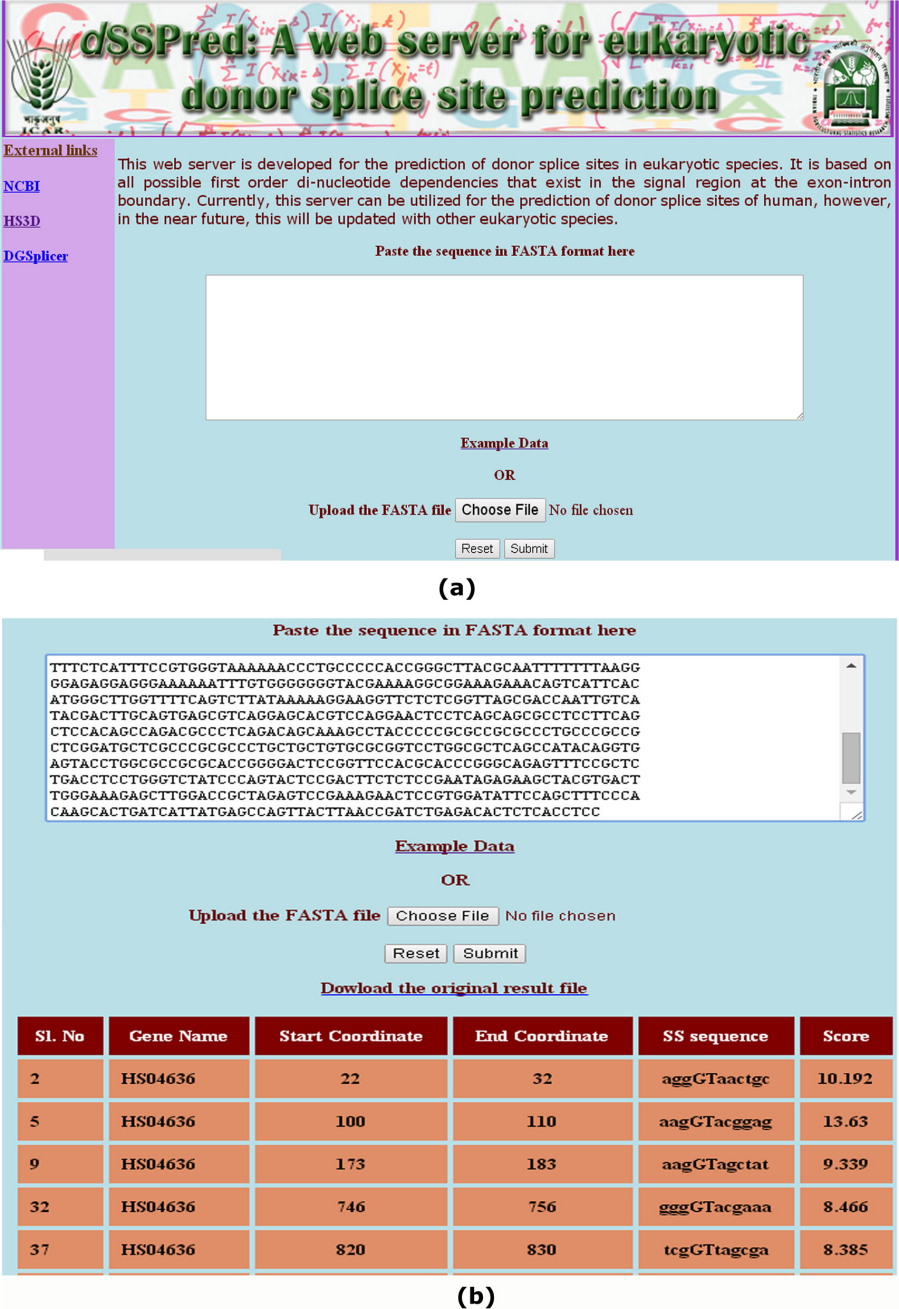
**Images of the developed web interface. (a)** home page and **(b)** result page of the prediction server. In the result page, gene name (2^nd^ column), start & end coordinates (3^rd^ and 4^th^ columns) of the predicted TSS on the gene, the sequences of 11 nucleotide TSS motif (5^th^ column) and score of being predicted as TSS (6^th^ column) are given.

The home page of the developed web server is shown in Figure 7(a) and the result page of the server after the execution of an example dataset is shown in Figure 7(b). A score is assigned to each predicted splice site and the splice site with the score of ≥7.16 is said to be predicted as true splice site. Further, higher the score more is the strength of the predicted splice site. In the result page, only the predicted true splice sites along with their corresponding score are displayed.

## Discussion

The process of splicing takes place in various steps being catalyzed by small nuclear ribonucleoproteins (snRNPs) that are the complex of snRNAs and proteins. The snRNPs are categorized into U1 snRNP, U2 snRNP, U4 snRNP, U5 snRNP and U6 snRNP based on the type of snRNA (U1, U2, U4, U5 and U6) associated with them [29]. Initially, the 5′splice site is recognized by U1 snRNP through the base pairing between U1 snRNA and the 5′ splice site [30]. On the other hand, the non-snRNP splicing factors interact with the 3′ splice site, resulting in the 5′ splice site being brought to the proximity of the 3′ splice site. Then, the U1/5′ splice site base pairing is weakened in an ATP-dependent step [31], allowing U2 snRNP to base pair with the branch site. Further, the U4/U5/U6 tri-snRNP complex is added, resulting in a noticeable destabilization of U1 snRNP from the spliceosome [32], followed by several rearrangements in which U1 is replaced by U5 and U6 at the 5′ splice site [33]. The U4/U6 base pairing within the U4/U5/U6 complex is disrupted; U4 is released from the spliceosome, and U6 snRNA base pairs with U2 snRNA [34]. These rearrangements finally allow the two constitutive catalytic steps to generate mature mRNA and release the intron.

Most of the existing methods for splice site prediction capture position specific signals as well as nucleotide dependencies. The pivotal role played by the nucleotide dependencies in the splice site motifs is explained by Roca *et al.* [35]. Therefore, the dependencies among nucleotides in the splice site motifs were accounted in the proposed prediction approach.

In splice site prediction using MLAs, the window sizes are generally determined through pilot studies *i.e.,* initially the prediction approach is tested with a small sample and window size is optimized on the basis of prediction accuracy and the final prediction is made on the determined window size. However, in our study the window size was determined through an analysis involving the associations among the nucleotides in the splice sites. Though MLAs has been successfully used in several approaches for the prediction of splice sites, the window lengths used are longer in size [16,18,20-23] and hence these approaches may not be appropriate for determining splice sites using short reads. But, our approach uses only 9 bp window length and may be more appropriate to identify the splice variants in short reads.

In MLAs, sometimes the parameters need to be optimized for a better training model (for example, the value of gamma in radial basis function while using SVM, number of units in the hidden layer while using multilayer perceptron neural network). However, in our approach, the only parameter need to be estimated is the threshold value and does not require extensive tuning like in MLAs. The value of the threshold is dependent upon the sensitivity and specificity and can be estimated in a single effort. Further, it will remain same with the change in degree of imbalanced-ness in the dataset.

From the sequence distribution analysis, it was found that there exist more than 70% similarity between TSS and FSS and the performance of the proposed approach was found better with the presence of this much (70%) similarity. Hence, it is expected that the proposed approach will perform better even in the presence of high percentage of similarity between the sequences of TSS and FSS. Further, the approaches were assessed using both redundant and non-redundant test dataset. It is inferred that the performance is better in presence of redundancy (Table 6) as compared to the dataset having no redundancy (Table 3).

The effect of window size on the performance of the proposed approach was determined by using other window sizes *i.e.*, 7 bp, 8 bp, 9 bp, 10 bp and 11 bp. The values of AUC-ROC and corresponding SE for different window sizes have shown that the window size of 9 bp is most preferable due to higher AUC-ROC and lowest SE (Table 1). In addition, the performance of the proposed approach was also assessed with different degrees of imbalanced-ness in the training dataset using ROC and PR curves. From ROC curves it was found that the performance is not affected by the presence of imbalanced-ness in the dataset (Figure 4a). However, it is seen that the PR curves are sensitive to the presence of imbalanced-ness in the dataset (Figure 4b).

To evaluate the performance of the proposed approach, a comparative analysis was carried out using non-redundant HS3D dataset with the existing approaches *i.e.,* MEM, MDD, WMM and MM1. The comparison was made in terms of AUC- ROC and AUC-PR curves. It was observed that the performance of the proposed approach is similar to that of MEM and MDD and better than that of WMM and MM1 (Table 3). Further, in another comparative analysis using redundant DGSplicer dataset, the performance of the proposed approach was found almost same with that of MEM and MDD but better than the other two approaches in terms of AUC-ROC and AUC-PR (Table 4). The earlier splice site prediction methods such as WMM and MDD have focused only on TSS and ignored the FSS to train the prediction model but FSS are also necessary in the prediction of splice sites [18]. Therefore, in our study, we have considered both the TSS and FSS for the donor splice site prediction. MEM with two point constraint (${S}_m^2$) and SAE resembles with each other as both consider di-nucleotide dependencies but these are two different prediction methods. Further, in MEM with two point constraint, not only the second order marginals are used but also the first order marginals with skip *0* (${S}_0^1$) are accounted as well, which is not the case in SAE and still the AUC-ROC and AUC-PR of SAE are at par with that of MEM. Moreover, MEM involves iterative procedure for optimization of different parameters as well as required 3 L number of more parameters to be estimated (for a sequence motif of length L) as compared to SAE, due to the involvement of 0^th^ order dependency (first order marginals with skip zero) in MEM. Whereas our approach is simpler because, it does not involve any iterative procedure as well as required less number of parameters to be estimated as compared to MEM. In MDD, higher order dependencies are taken into account but its performance is still similar to our approach. Moreover, the modeling of higher order dependency is sometime expensive due to more memory allocation as well as requires the estimation of large number of parameters.

The classifiers such as SVM, ANN and Classification tree have been successfully used in the area of splice site prediction, where the sequence data are first encoded in to numeric form and then used as input in such classifiers. Here, the term “encoding” is used in relation to the physical transformation of sequence dataset to numeric dataset, where the encoded dataset are further used as input in MLAs for prediction. In general, there is no disadvantage in encoding except that it takes one step more for encoding the sequence data into numeric form, which may require extra memory allocation. However, in SAE no such extra step is required and use of indicator variables is rather a simple representation of occurrence or non-occurrence of nucleotides, which is subsequently used (through expectation) for computation of probabilities of occurrence of nucleotides.

In MLAs, generally a model is defined that is function of certain parameters, where course search is performed over parameter space by the user to get a better training model. In this study, we are neither defining any such model nor optimizing any parameter to get a better training model. Further, the term “training dataset” is used here to refer the dataset used for finding the threshold value. Moreover, the dataset used for computing the threshold value can also be used as test dataset because threshold values are found almost unaltered under different proportion of TSS and FSS (dataset with different degrees of imbalanced-ness). However, in MLAs the optimum values of parameters may vary with respect to the size of the training set and the dataset used for training cannot be used as test dataset.

Even though, it has been suggested that a method that is able to capture higher order sequential relationships would perform better, its successful implementation is highly dependent on the availability of large dataset as they require the estimation of a large number of parameters [19]. In this investigation, only the first order dependencies among the bases were taken into account, which has also been the case for the MEM m2s5. However, in MEM m2s5, the first order marginals are also taken into account.

The proposed approach is based on the hypothesis if there exist associations among the nucleotide bases surrounding the splicing junction then the predictability of a base at any position, given any base at other position is higher in case of TSS as compared to the FSS. Hence, the SAE corresponding to a base given all other bases is less in case of TSS as compared to FSS. Since the proposed method will complement to other commonly used methods in prediction of donor splice sites with respect to shorter window size, it can contribute to the prediction of eukaryotic gene structure. In addition, the web server developed from this study will help enable the user for easy prediction of donor splice sites.

## Conclusions

An attempt is made to devise a simple procedure for the prediction of donor splice sites, which is based on di-nucleotide dependencies at all possible pairs of positions. This approach can be used for identifying the donor splice sites using the sequences of shorter window size. The proposed approach performs equally with MEM and MDD and better than WMM and MM1 and hence can be used as a complementary method to the existing methods in the prediction of eukaryotic gene structure.

## Availability

A user friendly web interface is available at http://cabgrid.res.in:8080/sspred for easy prediction of donor splice sites. The pre-processed HS3D dataset used in this investigation can be obtained from http://cabgrid.res.in:8080/sspred/dataset or http://bioinformatics.iasri.res.in/sspred_dataset.

## Additional file

Additional file 1:
**This file contain information regarding the results of threshold value for balanced situation under heading “Threshold value” and the threshold value for imbalanced situation under heading “Threshold value under imbalanced data”.**

## Abbreviations

WMM: Weighted matrix method
WAM: Weighted array model
MLAs: Machine learning approaches
ANNs: Artificial neural networks
SVM: Support vector machine
TSS: True splice site
FSS: False splice site
HS3D: Homo sapiens splice site dataset
bp: Base pairs
SAE: Sum of absolute error
MEM: Maximum entropy model
MDD: Maximal dependency decomposition
MM1: Markov model of first order
ROC: Receiving operating characteristics
AUC- ROC: Area under ROC curve
SE: Standard error
WL: Window length
PR: Precision-recall
AUC-PR: Area under PR curve
