# Supplementary material for: A statistical approach for 5′ splice site prediction using short sequence motifs and without encoding sequence data
Source: BMC Bioinformatics. 2014 Nov 25;15:362. doi: 10.1186/s12859-014-0362-6 (PMC4702320; doi:10.1186/s12859-014-0362-6)
Supplement: Additional file 1: — This file contain information regarding the results of threshold value for balanced situation under heading “Threshold value” and the threshold value for imbalanced situation under heading “Threshold value under imbalanced data”. [file 12859_2014_362_MOESM1_ESM.pdf]

Threshold value

Since the number of unique TSS for the 9bp window size is 1960, 60% of the TSS sequences *i.e.*, 1176 and an equal number of unique FSS were used in determination of threshold value. For performing the 10-fold cross validation, in each fold one-tenth of the data set *i.e.*, about 118 sequences from TSS and 118 sequences from FSS together were used as a test set and rest of the TSS and FSS datasets were used together as a training dataset. Figure S1 shows the graphical representation of the sensitivity, specificity and their difference for the 10 test sets corresponding to the 10-fold cross validation for different threshold values.

From the Figure S1, it is seen that the threshold values for different test sets are very close to each other. After taking average of the thresholds over the ten test sets, the final threshold value was obtained as -7.16 and was used further for the final prediction.

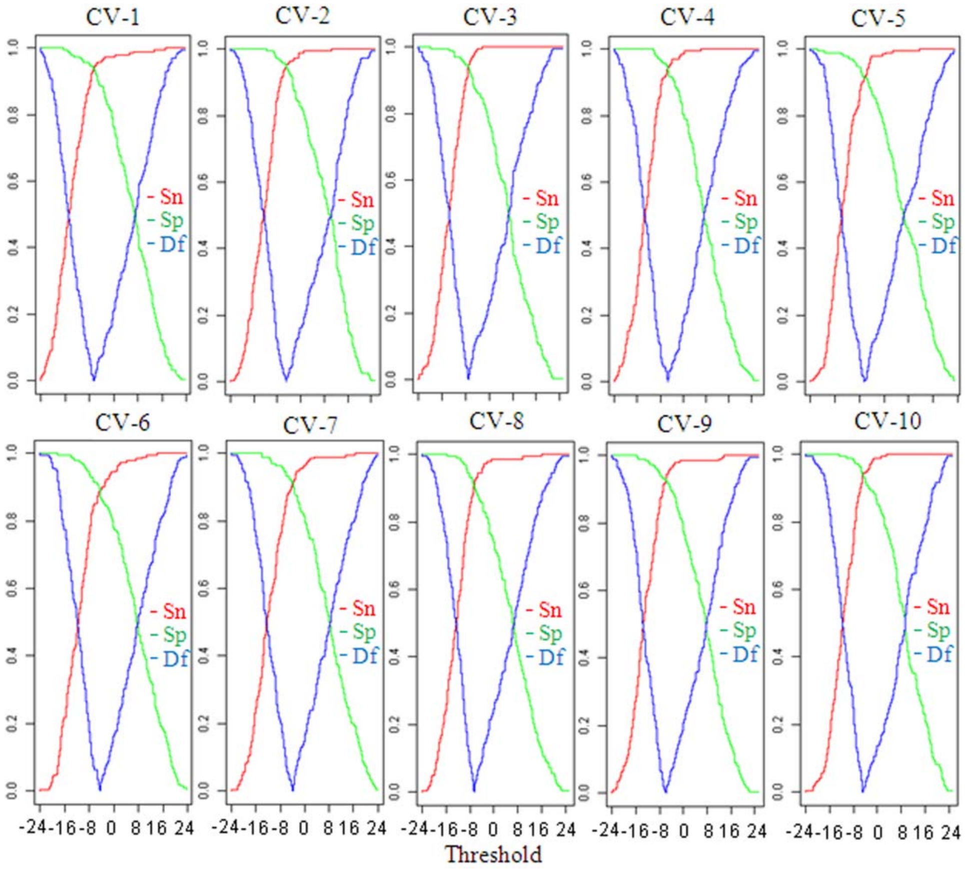

Figure S1. Sensitivity, specificity and their difference corresponding to different threshold values. For each fold in a 10-fold cross validation, Sensitivity (Sn), Specificity (Sp) and their difference (Df) are plotted against different threshold values. The value on the X-axis corresponding to the minimum Df is the determined threshold value for each test set.

Threshold value under imbalanced data

The values of the threshold obtained under different degree of imbalanced-ness for different window sizes are determined in the similar way as described in the main text for the balanced dataset and are shown graphically in Figure S2. It is observed from Figure S2 that though the values of the threshold varies within each imbalanced dataset for a given window size (corresponding to the 10 test dataset used in 10-fold cross validation), the average value of the threshold (black colored line) for the dataset with different degree of imbalanced-ness in a particular window size are very close to each other. This tendency shows the invariant nature of the threshold with different degrees of imbalanced-ness for a particular window size.

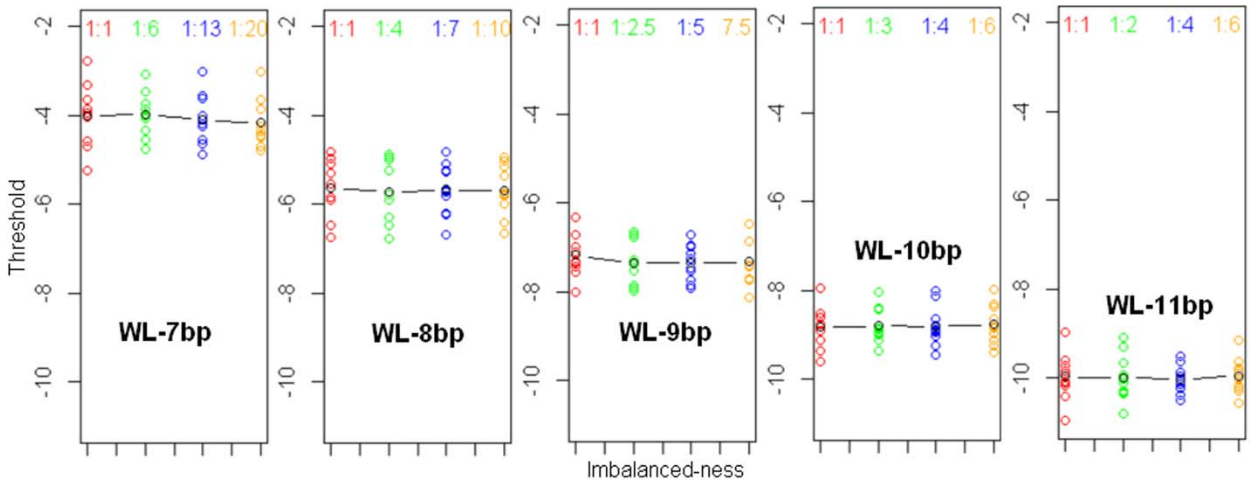

Figure S2. Threshold values under difference degrees of imbalanced-ness. For each window size, threshold values corresponding to 10-fold cross validation are plotted under different degree of imbalanced-ness. The proportion mentioned in the graph indicates the different degree of imbalanced-ness (different proportion of TSS and FSS) for different window sizes. The black color points indicate the average threshold value.
